# Impact of hybrid procedure on pulmonary arterial dimensions and right ventricular load after biventricular repair

**DOI:** 10.1186/s13019-023-02162-z

**Published:** 2023-02-07

**Authors:** Sebastian Uhl, Philippe Grieshaber, Raoul Arnold, Tsvetomir Loukanov, Matthias Gorenflo

**Affiliations:** 1 Department of Pediatric Cardiology/Congenital Cardiology, Heidelberg University Medical Center, Im Neuenheimer Feld 430, 69120 Heidelberg, Germany; 2 Cardiovascular Surgery Department, Heidelberg University Medical Center, Heidelberg, Germany

**Keywords:** Congenital heart defect, Right ventricle, Biventricular repair, Bilateral banding, Pulmonary arterial stenosis, Ductal stent, Hybrid procedure

## Abstract

**Background:**

Hybrid procedure with ductal stenting and bilateral pulmonary banding offers a temporary approach in high-risk neonates with complex congenital heart defects aiming biventricular repair. This procedure may also have negative impact concerning post-banding pulmonary stenosis resulting in right ventricular pressure load.

**Methods:**

Between 2010 and 2021 we identified 5 patients with interrupted aortic arch and complex congenital heart defect who underwent hybrid procedure and staged biventricular repair (“hybrid-group”). Other 7 cases with interrupted aortic arch were corrected in the neonatal phase without hybrid procedure (“nonhybrid-group”). Detailed intra- and extracardiac features and surgical procedures were documented as well as pulmonary interventions during follow up. Pulmonary vessel size was assessed by diameter of left and right pulmonary artery in absolute and indexed values. RV pressure was evaluated invasively via catheterization.

**Results:**

Survival in cases with hybrid procedure and staged biventricular repair was 91% for a follow-up time of 40.7 months (95% CI 26–55 months) and 100% in the non-hybrid-group. Postoperative results concerning left ventricular function showed normal LV dimensions and systolic function without relevant stenosis on distal aortic arch. Hybrid procedure was associated with impaired local pulmonary arterial diameter after debanding resulting in increased right ventricular pressure and need for interventions (number intervention per patient: hybrid group 1.7 ± 0.95, non-hybrid group 0.17 ± 0.41; *P* 0.003).

**Conclusions:**

Hybrid procedure in high-risk cases with interrupted aortic arch and staged biventricular repair shows good postoperative results with low perioperative mortality and normal left ventricular function. Due to potential risk of relevant pulmonary stenosis and right ventricular pressure load, follow up examinations must not only focus on left but also on the right heart.

**Graphical Abstract:**

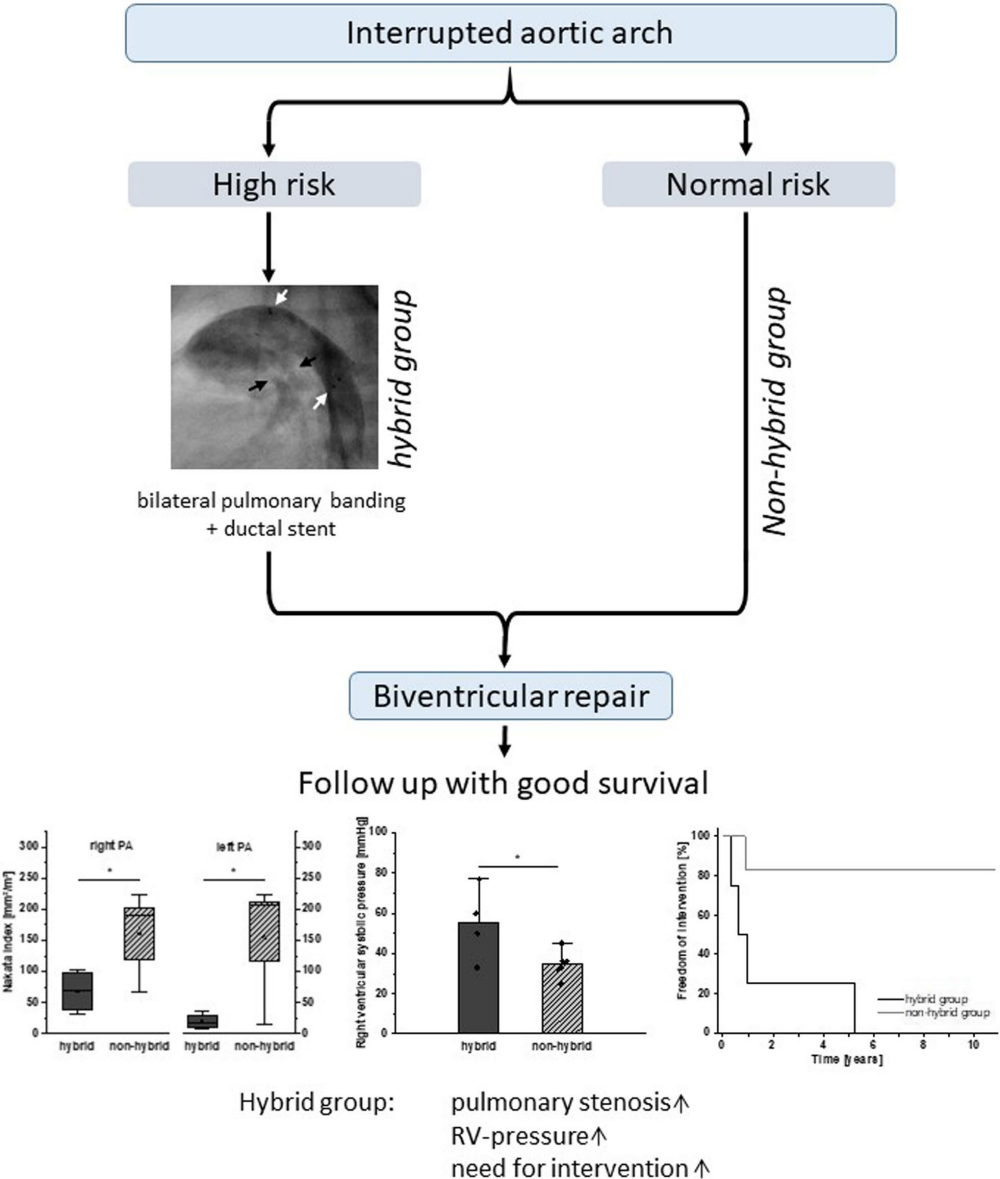

**Supplementary Information:**

The online version contains supplementary material available at 10.1186/s13019-023-02162-z.

## Introduction

In high-risk cases of neonates with complex congenital heart defect and ductus- dependent perfusion of the lower body, hybrid procedure (consisting of surgical bilateral banding of the pulmonary arteries (bPAP) and stenting of the patent ductus arteriosus) is described as alternative temporal procedure not only in univentricular anatomy of hypoplastic left heart, but also in selected cases with potential aim to biventricular repair [1–4]. In these cases, hybrid procedure is generally performed in the early postnatal period with following staged biventricular repair after the newborn phase. Advantages of this hybrid procedure without the side-effects of cardiopulmonary bypass (ischemic injury, inflammatory response) must be opposed to potential negative effects on pulmonary vascular growth with subsequent right ventricular pressure load and the need of re-interventions on pulmonary arteries [5].

In this study we focused on high-risk cases with interrupted aortic arch with staged biventricular repair after hybrid procedure. To investigate the impact of the hybrid procedure concerning pulmonary vessel growth and right ventricular pressure load, these cases were compared with cases of interrupted aortic arch anatomy with direct repair at newborn age.

## Methods

### Patient selection

Between 2010 and 2021, we identified 5 patients with complex congenital heart defect with interrupted aortic arch who underwent hybrid procedure and staged biventricular repair (“hybrid group”). These cases were statistically compared with 7 cases with interrupted aortic arch and immediate aortic repair in the newborn age (“non-hybrid group”). This study was performed as single center retrospective study at the University of Heidelberg, Germany and was approved by the local institutional ethic committee (S-224/2013, 20.12.2018) in accordance to the declaration of Helsinki and with consent of patient´s caregivers. Cases with univentricular palliation (e.g. hypoplastic left heart syndrome) were excluded from this study.

In literature, different risk scores were published to predict anatomic suitability for biventricular repair in neonates with aortic stenosis evaluating left ventricular dimensions, diameter of aortic valve and left ventricular outflow tract as well as aortic arch dimensions. We used Rhodes score [6], CHSS-1 [7], CHSS-2 [8] and Discriminant score [9] to compare anatomic complexity of left heart structures in our patient population as described in detail in literature.

The indication to surgical procedure (hybrid procedure versus immediate repair) was provided on individual patient´s case and in consent of pediatric and surgical team. Following indications existed in the hybrid-group: Patient 1: neonatal sepsis and intracardiac pathology with left ventricular outflow tract obstruction, hypoplastic aortic valve and ascending aorta, ventricular septal defect. Patient 2: neonatal sepsis, pulmonary steal with impaired perfusion of lower body and necrotizing enterocolitis. Patient 3: intracardiac pathology with left ventricular outflow tract obstruction, hypoplastic aortic valve and ventricular septal defect, persistent cardiac failure after reducing and re-escalation of prostaglandine-thearpy. Patient 4: cardiac failure with need of catecholaminergic treatment, pulmonary steal with impaired perfusion of lower body. Patient 5: premature hypotrophic twin with relevant intraventricular hemorrhage with recurrent seizures.

### Surgical and interventional procedures

Surgical and interventional approach for hybrid procedure and biventricular repair were investigated as well as interventional and surgical treatment on pulmonary vessels during follow up.

#### Bilateral pulmonary artery banding

Bilateral pulmonary artery banding (bPAB) was performed via a median sternotomy and a partial upper pericardiotomy. Both pulmonary arteries were banded to a diameter of 3.5 mm using PTFE (polytetrafluorethylene) bands as described previously [10]. The pericardium was completely closed after extensive irrigation with saline.

#### Ductal stent placement

Stenting of the patent ductus arteriosus (PDA) was performed percutaneous via catheter-intervention. Depending on the anatomical features of the PDA and vascular access (aortic, pulmonary), either pre-mounted balloon-expandable (Cordis® Genesis 10 × 19 mm, Terumo® Tsunami 6 × 18 mm) or self-expandable stents (Optimed® Sinus superflex DS, Germany, 7 × 20 mm, 7 × 15 mm) were implanted.

#### Percutaneous balloon-angioplasty of pulmonary arteries

After hemodynamic measurement and angiography of the pulmonary stenosis, a guidewire was delivered into the pulmonary artery and a balloon-angioplasty catheter was positioned (e.g. Cordis Powerflex® Pro PTA Dilatation catheter). Dilatation was performed between two to four time depending on interventional effect on the vessel. Paravasat was excluded by postinterventional angioplasty. For catheter based-intervention, femoral access was preferred.

#### Aortic arch reconstruction

After sternotomy and dissection of the heart and surrounding structures, cardiopulmonary bypass was established using arterial cannulation either directly in the aorta, the innominate artery or using a 3.5 mm PTFE-Shunt anastomosed to the innominate artery. An additional arterial cannula was placed in the PDA. The pulmonary artery bands were removed. After cooling to 18 to 24 °C, cardioplegic arrest was initiated. The VSD was usually approached using a transatrial approach and closed with xenopericardium using running sutures. The PDA was ligated and excised on the pulmonary side. If needed, the pulmonary bifurcation including the previous banding sites were now reconstructed using patch augmentation with equine xenopericardium (Matrix, AutoTissue, Berlin, Germany). For aortic arch reconstruction, either selective cerebral perfusion or deep hypothermic circulatory arrest was applied. The remnant stent material on the aortic site and all ductal tissue was removed. The descending aorta was now extensively mobilized and anastomosed with the proximal end of the aortic arch. Usually, the posterior wall was amenable to direct anastomosis while the anterior wall was augmented using homograft patch material.

### Invasive and non-invasive diagnostics

Non-invasive and invasive diagnostic procedures were performed with medical indication and retrospectively reviewed to investigate dimensions and function of the left ventricle as well as pulmonary arterial diameter and right ventricular pressure.

As additional follow up parameters survival, body weight and length, as well as echocardiographic marker for left ventricular dimensions and systolic function were investigated. Doppler measurement of maximal systolic velocity in der distal aortic arch was used as assessment for aortic pressure gradients. For diameter of left and right pulmonary artery, z-scores from Daubeney et al. were used [11]. To assess left ventricular outflow tract obstruction, we used calculation of Hirata et al., with cut-off of aortic valve annulus diameter in mm higher than body weight in kilogram + 1,5 [12]. Nakata index and lower lobe index were calculated as described in detail elsewhere [13–15].

### Statistical analysis

Statistical analysis and graphs were performed with OriginPro 2019. In text, absolute values are given as mean ± standard deviation and 95% coincidence interval (CI) as mentioned. Statistical analysis of two groups was performed by students t-test with *P* < 0.05 defined as significant. Box plot data is shown as median value as line with mean value as unfilled square and box as interquartile range. Whiskers indicate minimum and maximum range; black diamond-shaped dots show outliers. Bar graphs show mean value as bar with black diamond-shaped dots as single data point and whisker as minimum and maximum range. For equality test in Kaplan–Meier curve, log-rank test was performed with *P* < 0.05 defined as significant.

## Results

### Baseline demographics of patient population

Perinatal history, allometric data and the anatomical details of the congenital heart defect are shown in Table 1. There was a small statistical difference in birth age without a difference in birth weight between the two groups. We found no difference in the amount of cesarean section or prenatal diagnosis of the heart defect as possible cause for the difference in the birth age. Regarding intracardiac heart defect, left ventricular outflow tract obstruction was mainly found in the group with hybrid procedure as surrogate for a higher complexity of the cardiac anatomy in this group (P 0.02 vs non-hybrid group). To evaluate perioperative risks due to intracardiac anatomy, we evaluated several risk scores referring to left ventricular dimensions, outflow tract and aortic stenosis (Rhodes, CHSS-1, CHSS-2, Discriminant). Here we found comparable score values between hybrid group and non-hybrid group.

**Table 1:** Baseline demographics of patient population

	Hybrid group (n = 5)	Non-hybrid group (n = 7)	*P* value
Gestational age (week, mean ± SD)	37.8 ± 3.2	40.5 ± 1.4	0.033
Birth weight (kg, mean ± SD)	3.0 ± 0.6	3.3 ± 0.4	ns
Gender m/f	2/3	4/3	ns
Genetic syndrome n (%)	2 (40.0)	1 (14.3)	ns
Birth mode n spontaneous/caesarean section	3/2	2/5	ns
Prenatal diagnosis of heart defect n	1 (20)	1 (14.3)	ns
Preterm n (%)	1 (20.0)	0 (0)	ns
Cardiac structural diagnosis n (%)			
IAA Type A	2 (40.0)	5 (71.4)	ns
IAA Type B	3 (60.0)	2 (28.5)	ns
IAA Type C	0 (0)	0 (0)	ns
Aortic valve dysplasia	2 (40.0)	3 (42.9)	ns
Mitral valve dysplasia	1 (20.0)	1 (14.3)	ns
Ventricular septum defect	4 (80)	6 (85.8)	ns
Truncus arteriosus communis	1 (20.0)	0 (0)	ns
Aortopulmonary window	0 (0)	1 (14.3)	ns
Left ventricular outflow tract obstruction	3 (60.0)	0 (0)	0.02
Risk scores (mean ± SD)			
Rhodes score	− 0.59 ± 0.57	− 0.44 ± 0.45	ns
CHSS-1 score	− 10.5 ± 18.3	− 10.4 ± 9.3	ns
Discriminant score	− 0.17 ± 1.6	+ 0.12 ± 0.6	ns
CHSS-2 score	− 70 ± 16.4	− 53.5 ± 17.5	ns

*CHSS* Congenital Heart Surgeons' Society, *ns* not significant, *SD* standard deviation

### Preoperative conditions, operative variables and intra-hospital postoperative follow up

Preoperative clinical conditions and operative variables regarding the repair of the aortic arch are summarized in Table 2. In the hybrid group, different preoperative risks occurred leading to the indication for staged repair as mentioned in the method section. Hybrid procedure was performed at the mean age of 24.8 days (95% CI 9–40 days) and patients stayed on intensive care unit for a mean of 4.4 days (95% CI 2.9–5.9 days) after completion of the hybrid status. Hybrid status was maintained for a mean of 212 days (95% CI 175–258 days). During hybrid status, bilateral banding was dilated in 2 cases (1 × left pulmonary artery, 1 × left and right pulmonary artery). There was no need for re-intervention on PDA stent. No child died during hybrid status. Staged biventricular repair was performed at the age of 7.9 months (95% CI 6.0–9.7 m) with a mean weight of 6.7 kg (95% CI 6.2–7.1 kg). In 3 cases, pulmonary artery was reconstructed via Homograft patch, in 3 cases a balloon dilatation was performed simultaneously. In 1 case, a Contegra graft was implanted in pulmonary position to repair Truncus arteriosus communis. In the non-hybrid group, cardiac repair was performed at the mean age of 9 days (95% CI 5.4–12.8 days) and a mean weight of 3.3 kg (95% CI 3.0–3.6 kg). In this group, central pulmonary artery banding was implanted for staged closure of ventricular septal defect in 3 of 7 cases. In 1 case, right pulmonary artery was reconstructed for repair of an aortopulmonary window.

**Table 2:** Preoperative conditions and operative variables

	Hybrid group (n = 5)	Non-hybrid group (n = 7)	*P* value
Preoperative non-cardiac conditions n (%)			
Sepsis	1 (20.0)	0 (0)	ns
Cardiac shock	2 (40.0)	0 (0)	ns
Ischemic enterocolitis	0 (0)	1 (14.3)	ns
Intracranial hemorrhage	1 (20.0)	0 (0)	ns
Age at aortic arch repair (d, mean ± SD)	241 ± 58	9 ± 5	< .01
Weight at aortic arch repair (kg, mean ± SD)	6.7 ± 0.5	3.3 ± 0.4	< .01
Operative variables			
Cardiopulmonal bypass time (min, mean ± SD)	219 ± 59	141 ± 41	0.03
DHCA time (min, mean ± SD)	26 ± 39	35 ± 18	ns
ECMO postop n (%)	1 (20)	0 (0)	ns

*DHCA* deep hypothermic cardiac arrest, *ns* not significant, *SD* standard deviation

Operative variables for biventricular repair differed in between the groups with longer cardiopulmonary bypass time but comparable duration of deep hypothermic cardiac arrest in the hybrid group. Mean intensive care unit stay after repair of the interrupted arch was similar in hybrid group (16.5 days, 95% CI 8.5–24.4 days) and non-hybrid group (19.3 days, 95% CI 9.2–29.4 days; *p* 0.72). One child died several hours after biventricular repair caused by electromechanical decoupling without stabilization under extracorporeal life support (hybrid group). Other short-term complications were: need of implantation of permanent pace maker (1 patient in hybrid group), diaphragmic plication (1 patient in non-hybrid group), chylothorax (1 patient in non-hybrid group).

### Follow up: basic parameters and left ventricular characteristics

The follow up duration was comparable with 40.7 ± 14.9 months in the hybrid group (CI 26.1–55.4 m) and 61.6 ± 48.3 months in the non-hybrid group (CI 25.7–97.5 m; *p* 0.47). After hospital discharge, no patient died during follow up resulting in a survival rate of 4 of 5 patients in the hybrid group and 5 of 5 in the non-hybrid group.

Body growth in weight and length at the last follow up appointment was also comparable in both groups (percentile body length: hybrid group 16.3 ± 22.5, non-hybrid group 32.1 ± 40.4; p 0.50. Percentile body weight hybrid group 9.8 ± 8.7, non-hybrid group 34.5 ± 32.8; p0.18.) Left ventricular dimensions and dynamics were comparable in both groups (Table 3).

**Table 3:** Left ventricular dimensions and dynamics after biventricular repair

Echocardiographic parameter (mean ± SD)	Hybrid group (n = 4)	Non-hybrid group (n = 7)	*P* value
Left ventricular enddiastolic diameter, z-score	− 1.1 ± 1.6	− 0.1 ± 1.8	ns
Fractional shortening % (M-mode)	38.8 ± 12.7	40.1 ± 7.7	ns
Interventricular septum diastolic diameter, z-score	0.15 ± 1.43	1.39 ± 1.53	ns
Left ventricular posterior wall diastolic diameter, z-score	0.31 ± 0.99	1.09 ± 1.78	ns
Maximal velocity distal aortic arch (m/s)	1.94 ± 0.75	2.24 ± 0.89	ns

*SD* standard deviation

### Follow up: pulmonary arterial dimensions and right ventricular hemodynamics

**Fig. 1:**
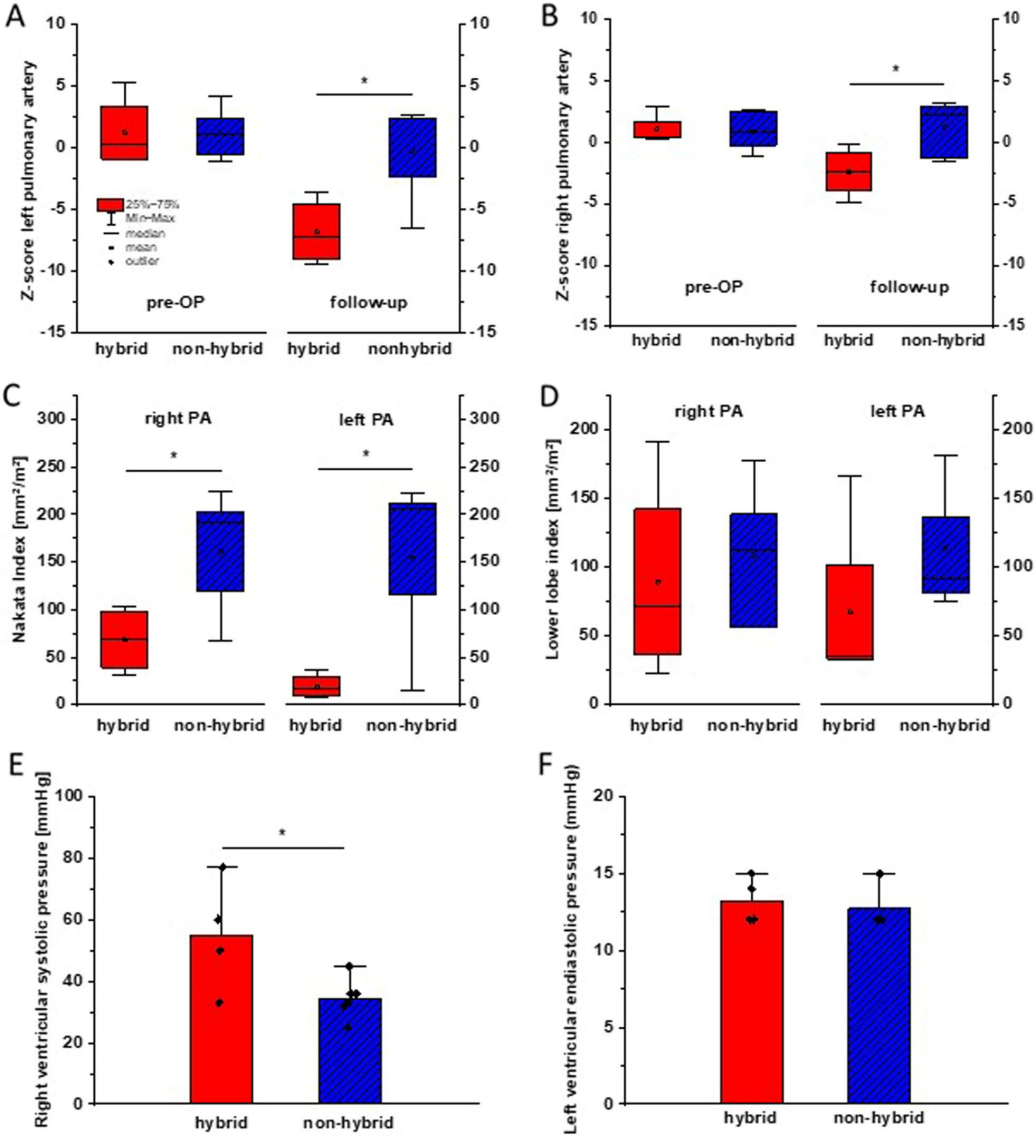
Impaired local pulmonary arterial size and increased right ventricular pressure after hybrid procedure and staged biventricular repair. Z-score of left (**A**) and right (**B**) pulmonary artery before and after cardiac repair. **C** Nakata index and **D** lower lobe index normalized to body surface area after biventricular repair. **E** Right ventricular systolic pressure after biventricular repair. **F** Left ventricular enddiastolic pressure after biventricular repair. PA pulmonary artery, *p < 0.05

After biventricular repair, we found significantly decreased local diameter of left and right pulmonary artery in the hybrid group compared to the non-hybrid group (Fig. 1A, B). As additional parameter to assess the size of the central pulmonary vascular bed we calculated Nakata index (referred to local vessel diameter) showing equivalent results with smaller values in the hybrid group (Fig. 1C). When evaluating peripheral vascular bed via lower lobe index, no such difference was observed (Fig. 1D).

Right ventricular pressure was evaluated invasively in 4 of 5 cases in the hybrid group and in 6 of 7 cases in the non-hybrid group. As shown in Fig. 1E, increased maximal systolic values were found in the hybrid group compared to non-hybrid group (hybrid group 55.0 ± 18.4 mmHg, non-hybrid group 34.5 ± 6.5 mmHg; *p* 0.034) as evidence for hemodynamic relevance of pulmonary stenosis. Distal pulmonary pressure was in a normal range in both groups excluding pulmonary hypertension as cause for increased RV pressure in hybrid group (distal mean PA pressure: hybrid group 13.0 ± 3.5 mmHg, non-hybrid group 14.2 ± 3.4 mmHg; *p* 0.61). Additionally, wedge pressure and left ventricular enddiastolic pressure were not different between the groups excluding an increased right ventricular pressure induced by left ventricular failure (Fig. 1F).

### Follow up: re-interventions on pulmonary vessels

**Fig. 2:**
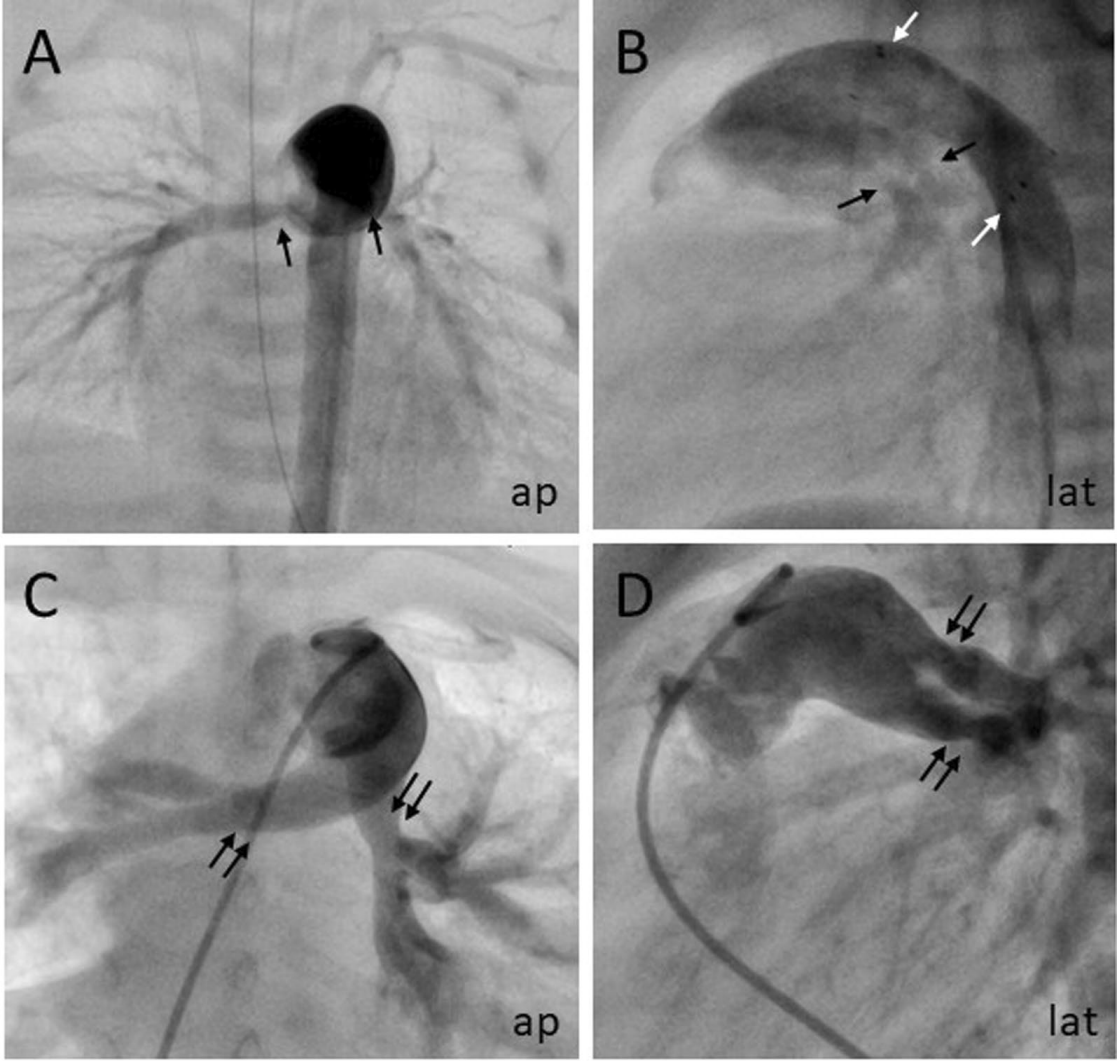
Pulmonary arterial conditions in a neonate with interrupted aortic arch during hybrid status and after staged biventricular repair. Angiography of pulmonary arteries with bilateral banding (single black arrows) in anteroposterior projection (**A**) and lateral projection (**B**) with stent in PDA (white arrows). **C**, **D** Bilateral pulmonary arterial stenosis (black double-arrows) eight months after debanding and staged biventricular repair with peak-to-peak gradient of 30–35 mmHg

Figure 2 shows pulmonary arterial conditions in a child during hybrid status with bilateral pulmonary banding and PDA stent (Fig. 2A, B) and eight months after staged biventricular repair with bilateral pulmonary arterial stenosis with a peak-to-peak gradient of 30–35 mmHg (Fig. 2C, D).

**Fig. 3:**
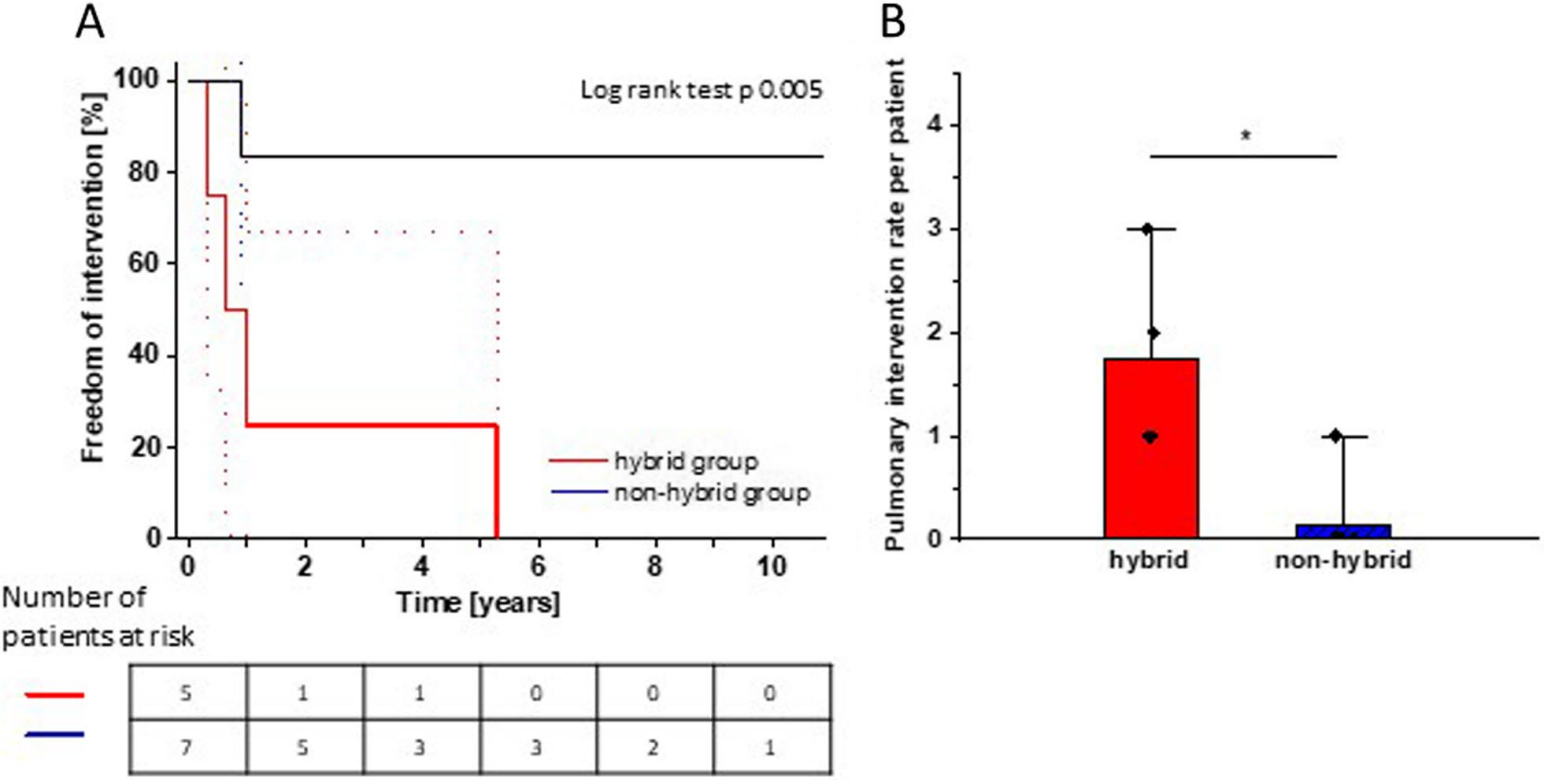
Increased need of catheter-based intervention on pulmonary stenosis after hybrid procedure and staged biventricular repair. **A** Kaplan–Meier curve with decreased time of freedom of intervention in hybrid group (*p* 0.005). **B** Number of interventions per patient. *p < 0.05

Freedom of intervention due to pulmonary arterial stenosis is presented as Kaplan–Meier curve in Fig. 3A with increased risk for relevant stenosis in the hybrid group (p 0.005 vs. non-hybrid group). As shown in Fig. 3B we also found an increased need of balloon-interventions in the hybrid group expressed as rate per patient (number of interventions per patient: hybrid group 1.7 ± 0.95, non-hybrid group 0.17 ± 0.41; *P* 0.003). Stent implantation in pulmonary vessels was not needed. In 1 case (hybrid group), surgical reoperation on pulmonary artery was performed. Further information in detail can be found in the Additional files 1 and 2: Tables S1 + S2).

## Discussion

Staged repair of complex congenital heart defects in combination with interrupted aortic arch and ductus-dependent perfusion of the lower body using hybrid procedure (bilateral pulmonary artery banding plus stenting of the patent ductus arteriosus) depicts an alternative approach in high-risk cases aiming biventricular repair. Whereas there are several publications reporting survival and re-intervention rate after hybrid procedure in univentricular palliation [2, 16, 17]), the follow up in children with biventricular repair after hybrid procedure and the need of reinterventions after debanding is not reported in detail yet. Erek et al. [18] published a case report of six children with different aortic arch pathology with left ventricular outflow obstruction and biventricular repair with follow up until hospital discharge. A study of Kapravelou et al. [19] present hybrid procedure in three cases with IAA and biventricular repair with a maximum follow up in one case of three years. Lee et al. [20] describe a case report of one child with microdeletion 22q11 with IAA and left ventricular outflow tract obstruction with cardiac repair after 10 months after hybrid procedure with a postoperative follow up of 56 days. Yerebakan et al. [2] report cases with staged biventricular repair after hybrid procedure focusing on left ventricular anatomy and hemodynamic.

In our study, we focused on the impact of hybrid procedure on pulmonary artery and right ventricle in cases with IAA after staged biventricular repair. These results were compared with a group of neonates with IAA arch repaired one-stage with a mean follow up of 3.4 years, in which we observed a good survival in both groups. By excluding univentricular palliation after hybrid procedure and cases with critical aortic coarctation with hybrid procedure we ensured a comparability between the hybrid group as “intervention group” and non-hybrid group as “control group” concerning operative strategies and hemodynamic evaluation on right ventricle. There are different risk scores published to evaluate feasibility for biventricular repair in neonates with aortic stenosis. We used these scores not to decide between uni- or biventricular approach but to compare our groups concerning intracardiac anatomic complexity. Here we found comparable score values.

Follow up examinations in the hybrid group after staged repair showed normal left ventricular function with normal wall thickness and comparable echocardiographic parameters assessing pressure gradients in the aortic arch.

Notably, local pulmonary arterial diameter and Nakata indices (as size parameter for central vascular tree) were impaired after debanding of the bilateral pulmonary banding. Consecutively, we found higher pressure in right ventricle in this group confirming the hemodynamic relevance of the pulmonary stenosis. Subsequently the need for pulmonary catheter-intervention was higher after staged biventricular repair with hybrid procedure compared to immediate reconstruction of the aortic arch without bilateral pulmonary banding. Pulmonary hypertension as cause for increased RV pressure in the hybrid group was excluded as well as left ventricular backward failure. Similar effects on pulmonary vessels were observed in publications with univentricular palliation after hybrid procedures [5, 16]. In contrast to these findings, lower lobe index as parameter to assess peripheral pulmonary arterial vascular bed was not reduced. A possible interpretation for this finding is an impaired vessel growth at the local site of the former banding, whereas the peripheral vascular bed itself is not affected. Notably, in most cases residual pulmonary arterial stenosis was accessible to catheter-based intervention without need of re-operation or increased perinterventional morbidity relativizing this potential disadvantage of the hybrid procedure in high-risk cases.

### Study limitation

This study is a retrospective review in a single center and the patient population differ concerning preoperative risk factors (affecting indication towards hybrid procedure). The allocation into the two compared groups (hybrid procedure versus immediate repair of interrupted aortic arch) was done retrospectively and without randomization. Hybrid procedure was performed in high-risk cases whereas single stage-repair was done in children with lower risk profile. However, this bias will not influence the inter-group comparability concerning pulmonary arterial growth, because single-stage repair does not include surgery on pulmonary vessels by default. Further studies are needed to clarify whether the pulmonary stenosis after debanding is induced by the surgical impact itself (banding and debanding) or by the duration of the banding status, especially as there is evidence in other publications that findings in univentricular hearts may differ from cases after biventricular repair after hybrid procedure [5], potentially induced by hemodynamic difference between biventricular condition and after univentricular palliation with non-pulsatile low pressure in pulmonary artery.

## Conclusion

For high-risk cases of complex congenital heart defects with interrupted aortic arch, hybrid procedure can be performed for staged biventricular repair with a good survival rate. Potential advantages (cardiac bypass operation after critical postnatal period and after stabilization of extracardiac pathologies) must be weight up with potential disadvantages (impaired local pulmonary arterial growth and increased right ventricular load after debanding of the bilateral banding) and decided individually on patient´s case and history.

## Supplementary Information

**Additional file 1: Table S1.** Additional case information Hybrid group.

**Additional file 2: Table S2.** Additional case information Non-hybrid group.

## Data Availability

The datasets used and analysed during the current study are available from the corresponding author on reasonable request.

## Abbreviations

CI: Confidence interval
bPAB: Bilateral pulmonary banding
ECMO: Extracorporeal membrane oxygenation
IAA: Interrupted aortic arch
LV: Left ventricle
PDA: Patent ductus arteriosus
PTA: Percoutaneous transluminal angioplasty
PTFE: Polytetrafluorethylene
RV: Right ventricle
SD: Standard deviation
VSD: Ventricular septal defect
